# Plasma protein expression profiles, cardiovascular disease, and religious struggles among South Asians in the MASALA study

**DOI:** 10.1038/s41598-020-79429-1

**Published:** 2021-01-13

**Authors:** Long H. Ngo, M. Austin Argentieri, Simon T. Dillon, Blake Victor Kent, Alka M. Kanaya, Alexandra E. Shields, Towia A. Libermann

**Affiliations:** 1 Harvard Medical School, Harvard University, 1309 Beacon Street, Brookline, MA 02447 USA; 2 Department of Medicine, BIDMC Genomics, Proteomics, Bioinformatics, and Systems Biology Center, Beth Israel Deaconess Medical Center, Boston, USA; 3 Department of Biostatistics, Harvard T.H. Chan School of Public Health, Boston, USA; 4 Department of Medicine, Harvard/MGH Center On Genomics, Vulnerable Populations, and Health Disparities, Mongan Institute, Massachusetts General Hospital, Boston, USA; 5 School of Anthropology and Museum Ethnography, University of Oxford, Oxford, UK; 6 Department of Sociology, Westmont College, Santa Barbara, USA; 7 Department of Medicine, University of California, San Francisco (UCSF), San Francisco, USA

**Keywords:** Proteins, Proteomics, Cardiovascular diseases, Biomarkers, Risk factors

## Abstract

Blood protein concentrations are clinically useful, predictive biomarkers of cardiovascular disease (CVD). Despite a higher burden of CVD among U.S. South Asians, no CVD-related proteomics study has been conducted in this sub-population. The aim of this study is to investigate the associations between plasma protein levels and CVD incidence, and to assess the potential influence of religiosity/spirituality (R/S) on significant protein-CVD associations, in South Asians from the MASALA Study. We used a nested case–control design of 50 participants with incident CVD and 50 sex- and age-matched controls. Plasma samples were analyzed by SOMAscan for expression of 1305 proteins. Multivariable logistic regression models and model selection using Akaike Information Criteria were performed on the proteins and clinical covariates, with further effect modification analyses conducted to assess the influence of R/S measures on significant associations between proteins and incident CVD events. We identified 36 proteins that were significantly expressed differentially among CVD cases compared to matched controls. These proteins are involved in immune cell recruitment, atherosclerosis, endothelial cell differentiation, and vascularization. A final multivariable model found three proteins (Contactin-5 [CNTN5], Low affinity immunoglobulin gamma Fc region receptor II-a [FCGR2A], and Complement factor B [CFB]) associated with incident CVD after adjustment for diabetes (AUC = 0.82). Religious struggles that exacerbate the adverse impact of stressful life events, significantly modified the effect of Contactin-5 and Complement factor B on risk of CVD. Our research is this first assessment of the relationship between protein concentrations and risk of CVD in a South Asian sample. Further research is needed to understand patterns of proteomic profiles across diverse ethnic communities, and the influence of resources for resiliency on proteomic signatures and ultimately, risk of CVD.

## Introduction

South Asians experience a higher burden of cardiovascular disease (CVD) compared with other U.S. populations; the majority of this increased CVD is likely due to modifiable risk factors^1^. While psychosocial stress has been previously demonstrated to be an important factor associated with risk of CVD^2–4^, there is a paucity of research on whether these factors associate with CVD risk in South Asian populations^1^. Stress has nonetheless been identified as one of the top ten drivers of health disparities worldwide by the World Health Organization, with significant disparities in exposures to stress between majority versus minority racial/ethnic and low socioeconomic groups^5^. At the same time, there are also few studies assessing resources for resilience that may mitigate risk of CVD among U.S South Asians.

Religion and spirituality (R/S) are particularly understudied psychosocial factors that may be important sources for resilience, support, and social engagement—all factors that might attenuate the adverse impacts of stress and improve health^6–10^. In some cases, such as when an individual understands a stressful life situation as proof that they are being punished for their transgressions or abandoned by God. However, R/S beliefs may compound the negative impact of stress and increase risk of disease^11,12^. R/S practices and beliefs are also especially important in many U.S. minority communities. For example, while 77% of the U.S. general population reported in 2014 that religion is very or somewhat important to them, this number was 91% and 84% among African Americans and Hispanics/Latinos, respectively^13^. National data specific to South Asians in the U.S. have not typically been available, but recent research conducted among South Asian participants from the Mediators of Atherosclerosis in South Asians Living in America (MASALA) Study who also are enrolled in the Study on Stress, Spirituality, and Health (SSSH), showed that 29% of South Asian participants reported engaging in group prayer several times a month or more, and 59% reported attending religious services 2–3 times per month or more^14^. Religious organizations play a pivotal role in identity maintenance and formation of immigrant populations, and many U.S. South Asians look to their religious communities for a sense of identity and belonging^15^. Crucially, not only do religious communities provide resources for well-being, but religious beliefs also function as strategies for resilience, particularly in successfully coping with stressful situations^16^. Recent research among MASALA participants in the SSSH has also found that high levels of self-rated R/S are associated with better self-rated health^14^, and that self-identifying as a very religious or spiritual person and positive religious coping is beneficially associated with self-rated health, emotional functioning, and anxiety, after controlling for the influence of other religious beliefs and practices^17^.

The South Asian population has been neglected in much of the extant literature documenting associations between R/S and incident CVD^8,18–21^, and no work has sought to identify the biological pathways or mechanisms through which R/S might affect risk of developing CVD in this population. One such area that holds particular promise for advancing knowledge regarding CVD risk and the ways that religion and spirituality might modify this risk is the field of proteomics^22^. Protein biomarkers are particularly effective in assessing CVD risk, as they carry information on pathophysiological status^23^, with many studies published that have identified protein biomarkers demonstrating predictive associations with CVD risk in other populations^24–27^. While existing CVD proteomics research has been conducted in white and high-risk clinical populations, there is currently a great need to investigate whether these findings are robust among different racial/ethnic populations in the U.S. who experience a disproportionate burden of CVD—in this case, South Asians. No proteomics study to date has been conducted in the U.S. South Asian community.

In this exploratory study, we begin to address these gaps in the literature by assessing the influence of proteomic expression profiles on risk of CVD, and subsequently assessing the potential modifying effect of select measures of R/S, among a subset of U.S. South Asians from the MASALA study who are participating in the SSSH. Our study leverages the strengths of the SOMAscan analysis platform, which is an aptamer-based, multiplex, highly sensitive, affinity proteomics platform that simultaneously quantifies 1,305 biologically-relevant human proteins in serum, plasma, CSF, other bodily fluids, cells, and tissues^28–32^. Specifically, we investigate plasma protein signatures among 50 MASALA participants with incident CVD and 50 sex- and age-matched controls. In this paper, we present the first proteomics analyses of protein levels in relationship to CVD events within a U.S. South Asian population, as well as the first assessment in this population of the R/S influences on risk of CVD either directly, or by modifying significant protein-CVD associations. To our knowledge, this is the first study to analyze proteomics signatures in relationship to measures of R/S for any population.

## Results

### Sample characteristics

**Figure 1:**
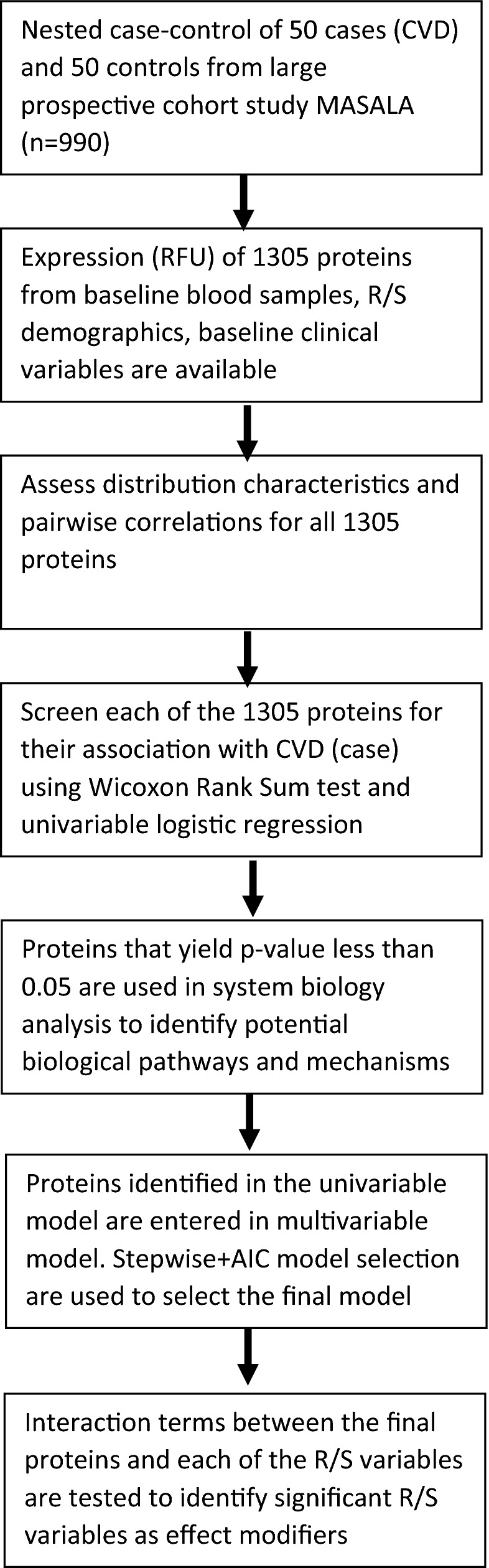
Analysis flow diagram.

Figure 1 presents a flow diagram of each step in the analysis. The study sample characteristics for the 50 incident CVD cases and 50 controls are reported in Table 1. The prevalence of smoking was low at 5%. Participants with CVD events had a much higher prevalence of diabetes (48% versus 26%), higher CAC scores (median 256 versus 8), higher LDL (median 113 versus 93 mg/dL), and lower HDL-cholesterol (median 45 versus 48 mg/dL).

**Table 1:** Sample characteristics of the MASALA case–control sample, 2010–2018^*^.

	Full sample (N = 100)	Cases with CVD (N = 50)	Controls without CVD (N = 50)	P-value^#^
Age, years	64.4 (7.4)^†^ 65 (60–69)	64.4 (7.3) 65 (60–69)	64.4 (7.5) 65 (59–69)	0.967
Female	22%	22%	22%	1.000
Smoking	5%	6%	4%	0.646
Diabetes	37%	48%	26%	0.023
Hypertension	61%	64%	58%	0.539
CAC Score	310 (496) 61 (0–465)	446 (512) 256 (24–744)	174 (444) 8 (0–136)	0.0001
LDL (mg/dL)	106 (33) 99 (82–131)	111 (33) 113 (91–142)	101 (32) 93 (81–116)	0.046
HDL (mg/dl)	50 (15) 47 (40–57)	48 (13) 45 (39–55)	51 (16) 48 (40–60)	0.351

*Match factors: Age and Sex.

^†^First row is mean and standard deviation. Second row is median and range (25^th^-75^th^ percentile).

^#^Wilcoxon Rank Sum or Chi-square test.

### Protein characteristics

**Figure 2:**
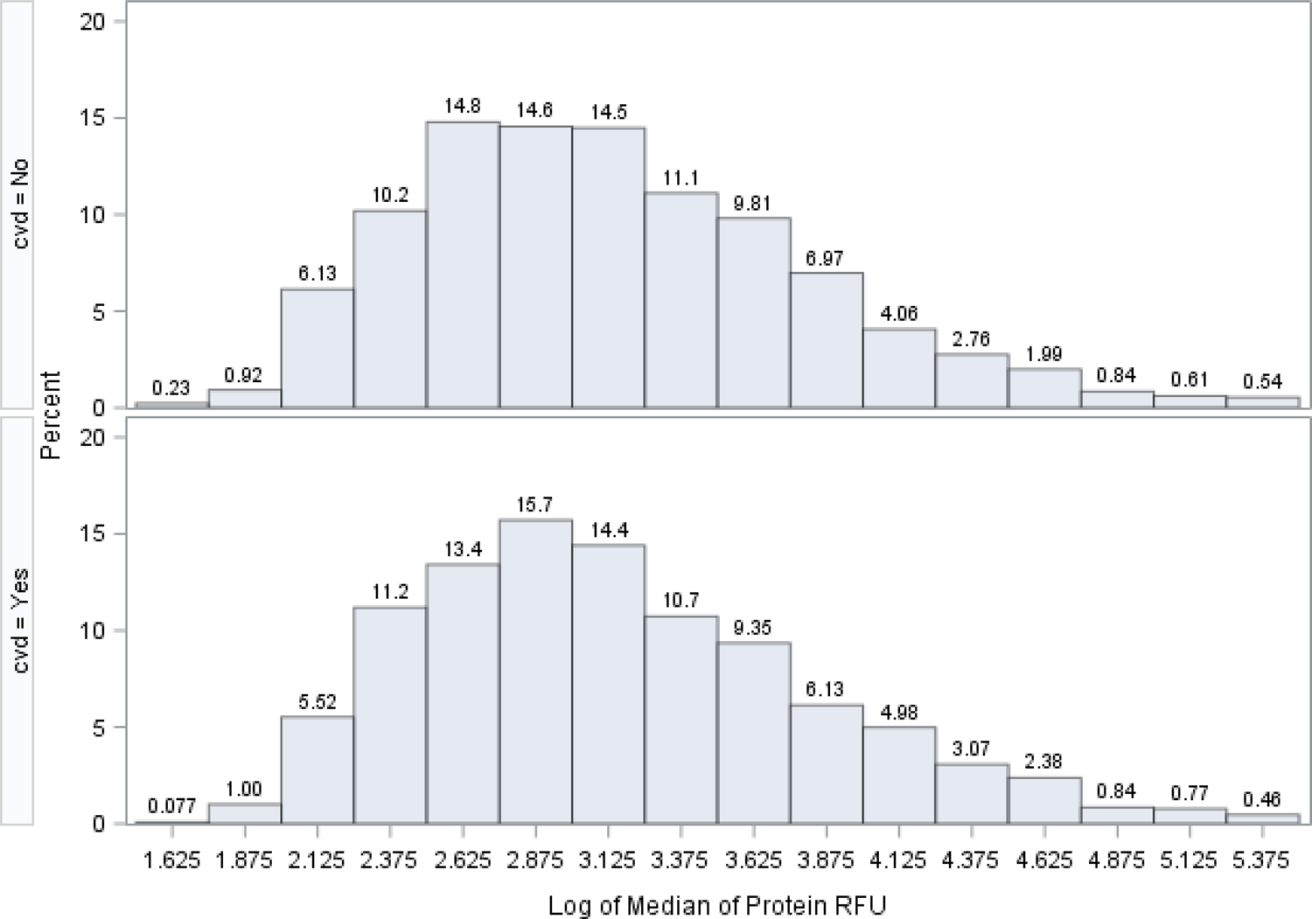
Distribution of the log base 10 of 1305 median estimates from 1305 proteins. Distribution of the log base 10 of 1305 median estimates from 1,305 proteins.

**Figure 3:**
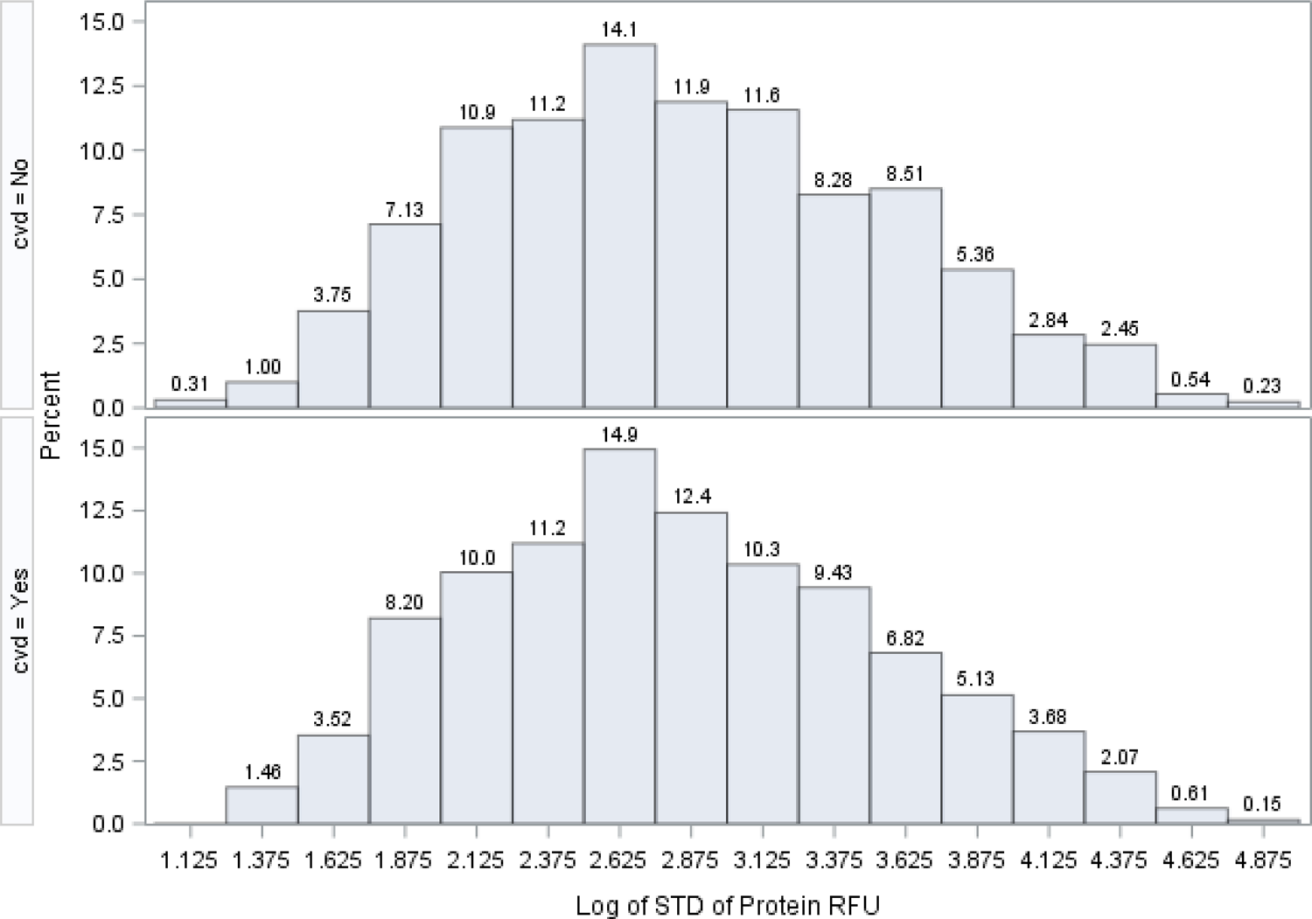
Distribution of the log base 10 of 1305 standard deviation estimates from 1305 Proteins.

Figure 2 shows the distribution of the medians (from all patients) for all 1305 SOMAscan proteins on a logarithm base 10 scale. The protein relative fluorescence units (RFU) magnitude ranged from 1.6 logs to nearly 5.4 logs. Proteins with low RFU values (2 logs or below; RFU < 100) comprised about 6.5% of all proteins (85 out of 1,305 proteins). The protein with the largest RFU value was Apolipoprotein E (isoform E4), with a median value of 237,512 RFU, and the lowest RFU value protein was Baculoviral IAP repeat-containing protein 7 Isoform beta, which had a median RFU level of 53. The distribution of the median estimates for all proteins was similar both in those with and without a CVD event. The variability of these protein RFUs is shown in Fig. 3, where the logarithm base 10 of the standard deviation is displayed. These values ranged from 1.1 logs to 4.8 logs.

**Figure 4:**
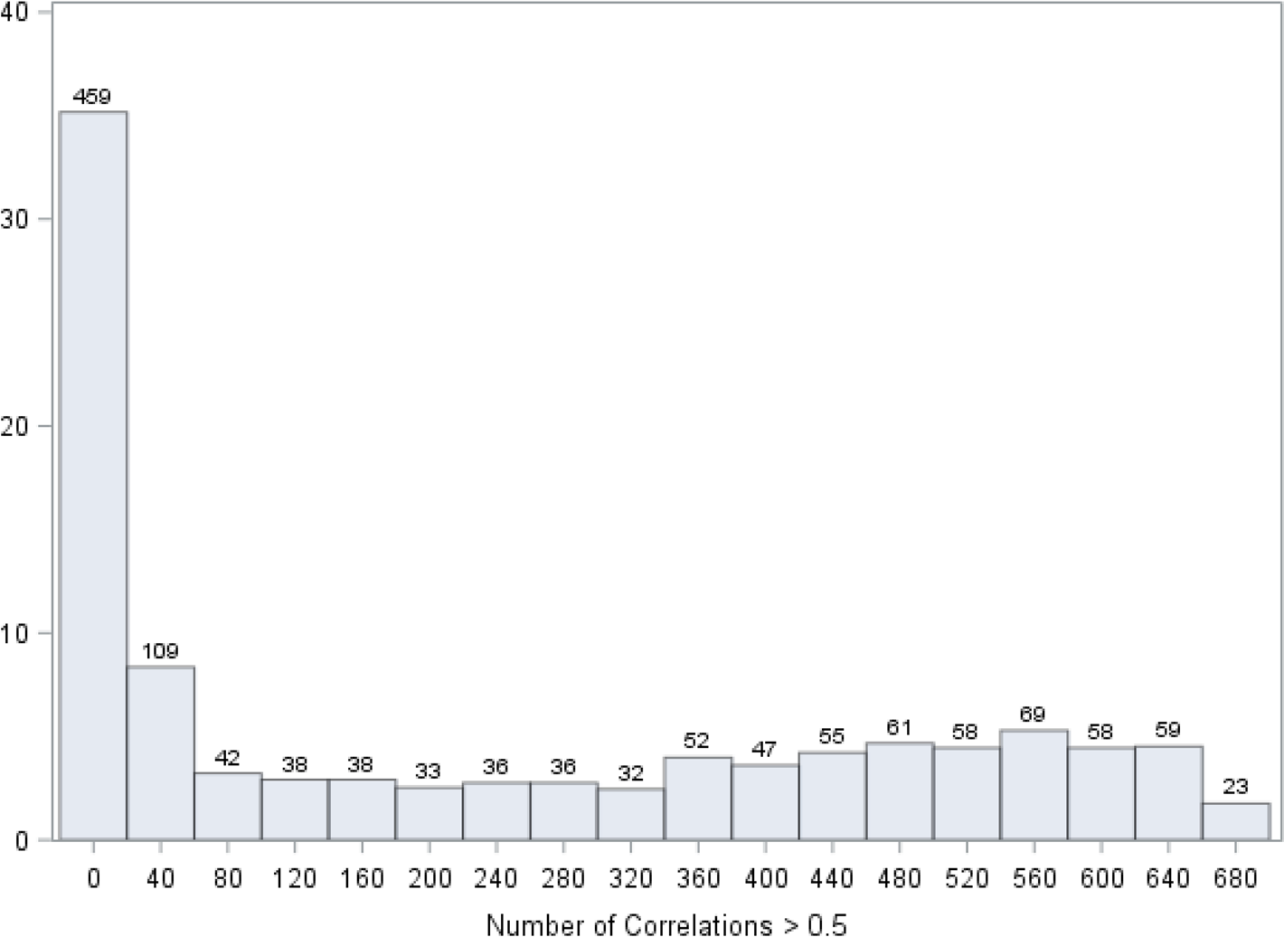
Distribution of the number of proteins that correlate with other proteins at the Spearman correlation coefficient of 0.5 or above. The purpose of this correlation analysis is to identify proteins that are least correlated to other proteins. For example, in the first bar of this graph, there are 459 proteins that are correlated at 0.5 or above with 0 to 20 other proteins. There are 23 proteins that are highly correlated (0.5 or above) with 660 to 700 other proteins.

Of these 1305 proteins, we expected many to be correlated. In selecting our final set of protein biomarkers to use in final multivariable models, we therefore preferentially chose proteins with little or no correlation to other proteins. We performed a Spearman rank correlation analysis on all 1,305 proteins, which yielded 1,703,025 correlation coefficients. Figure 4 shows the distribution of protein–protein correlations at the Spearman correlation coefficient of 0.5 or above. There were 459 proteins that correlated at 0.5 or above with 0–30 other proteins. There were also 23 proteins that correlated at 0.5 or above with 660–700 other proteins, representing redundant information. In cases where our analyses yielded two proteins with an equal effect on CVD, we selected the protein correlated with fewer other proteins and thus having more independent information (i.e., located toward the left side of the x-axis in Fig. 4).

### Protein associations with CVD

Screening for proteins associated with CVD incidence was based on two univariable statistical tests: the Wilcoxon Rank Sum test (not shown), where we do not make assumptions about the distribution of protein RFU and the rank of the RFU data was used; and univariable logistic regression. Table 2 shows three key columns: The Spearman correlation of each protein with CVD, the number of other proteins correlated with the index protein at 0.5 or higher, and the p-value from the logistic regression model for each protein’s association with CVD. We used results from the logistic regression to guide our screening of each individual protein, and the Wilcoxon Rank Sum test p-value as a second check. Using this method, we identified 36 proteins that met the 0.05 type-I error threshold (Table 2). Of these 36 proteins that were identified as significant by logistic regression, 32 were also identified as significant by the Wilcoxon Rank Sum test (2 of the 4 have borderline significant p-values). Six proteins showed up-regulated associations with CVD incidence (i.e., increased protein levels are associated with higher CVD risk), and 30 showed down-regulated associations (i.e., decreased protein levels are associated with higher CVD risk). Proteins shown in Table 2 are sorted by the p-values of the logistic regression models and ranked from the smallest p-value (Contactin-5, p-value = 0.0051) to the largest p-value that is still less than 0.05 (Cystatin-SA, p-value = 0.049).

**Table 2:** Univariable logistic regression for the selection of top 36 proteins (p-value <  = 0.05) associated with incident CVD.

Protein full name	Entrez Gene Symbol	Correlation with Incident CVD	No. of proteins corr. > 0.5	Logistic Reg P-value
Contactin-5	CNTN5	− 0.332	76	0.0051
Tyrosine-protein kinase transmembrane receptor ROR1	ROR1	− 0.268	378	0.0111
Ectodysplasin-A, secreted form	EDA	− 0.251	427	0.0116
Low affinity immunoglobulin gamma Fc region receptor II-a	FCGR2A	0.185	1	0.0138
Leucine-rich repeats and immunoglobulin-like domains protein 3	LRIG3	− 0.275	650	0.0138
Superoxide dismutase [Mn], mitochondrial	SOD2	− 0.31	196	0.0150
Cadherin-5	CDH5	− 0.245	262	0.0157
Cystatin-M	CST6	− 0.243	23	0.0158
Natural cytotoxicity triggering receptor 1	NCR1	− 0.243	519	0.0168
Tyrosine-protein kinase receptor Tie-1, soluble	TIE1	− 0.235	310	0.0169
Matrilin-2	MATN2	− 0.249	537	0.0226
Coiled-coil domain-containing protein 80	CCDC80	− 0.215	30	0.0227
AT-rich interactive domain-containing protein 3A	ARID3A	0.267	551	0.0228
Ck-beta-8–1	CCL23	− 0.285	535	0.0242
Neuronal growth regulator 1	NEGR1	− 0.268	480	0.0256
Granzyme A	GZMA	− 0.312	424	0.0272
SLIT and NTRK-like protein 5	SLITRK5	− 0.257	483	0.0301
Mast/stem cell growth factor receptor Kit	KIT	− 0.238	493	0.0314
Casein kinase II 2-alpha':2-beta heterotetramer	CSNK2A2 CSNK2B	0.252	498	0.0322
Cysteine-rich with EGF-like domain protein 1	CRELD1	− 0.212	68	0.0329
GDNF family receptor alpha-1	GFRA1	− 0.215	450	0.0334
Mannan-binding lectin serine protease 1	MASP1	− 0.236	570	0.0340
Complement factor B	CFB	− 0.143	1	0.0350
Protein-glutamine gamma-glutamyltransferase E	TGM3	− 0.098	1	0.0381
Interferon gamma receptor 1	IFNGR1	− 0.273	290	0.0393
Interleukin-1 receptor type 2	IL1R2	− 0.207	374	0.0402
Tumor necrosis factor ligand superfamily member 18	TNFSF18	− 0.254	161	0.0404
A disintegrin and metalloproteinase with thrombospondin motifs	ADAMTS13	− 0.236	333	0.0406
Apolipoprotein M	APOM	− 0.23	599	0.0417
Low affinity immunoglobulin epsilon Fc receptor	FCER2	− 0.128	11	0.0425
Serine/threonine-protein kinase receptor R3	ACVRL1	0.265	160	0.0429
Neurogenic locus notch homolog protein 1	NOTCH1	− 0.224	635	0.0433
Carbonic anhydrase 6	CA6	− 0.22	12	0.0469
Lamin-B1	LMNB1	0.229	543	0.0469
Interleukin-18 receptor accessory protein	IL18RAP	0.204	456	0.0471
Cystatin-SA	CST2	− 0.18	195	0.0485

Correlation with Incident CVD is calculated using the Spearman correlation coefficient between each protein and Incident CVD. The # proteins corr > 0.5 indicates the number of other proteins that correlate with this protein at 0.5 or higher. Logistic Regression p-value is Wald chi-square p-value of univariable logistic regression modeling incident CVD.

### Systems biology analysis

**Figure 5:**
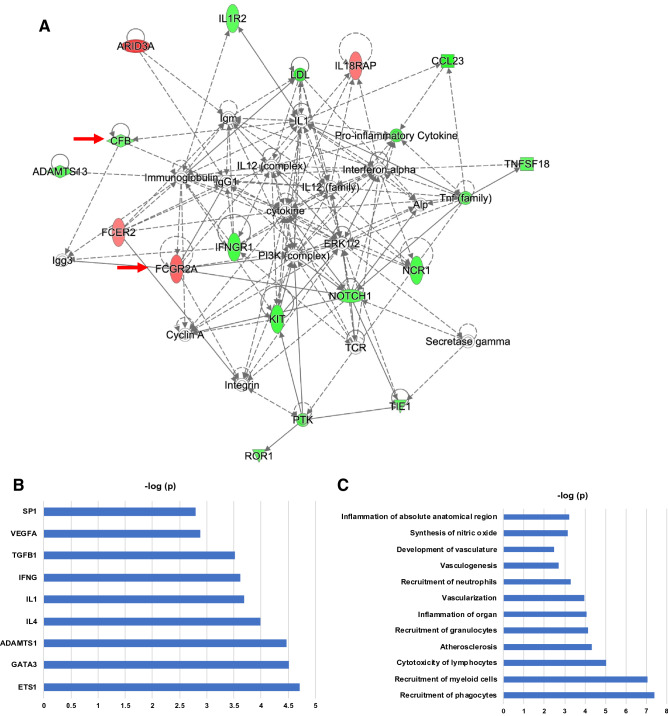

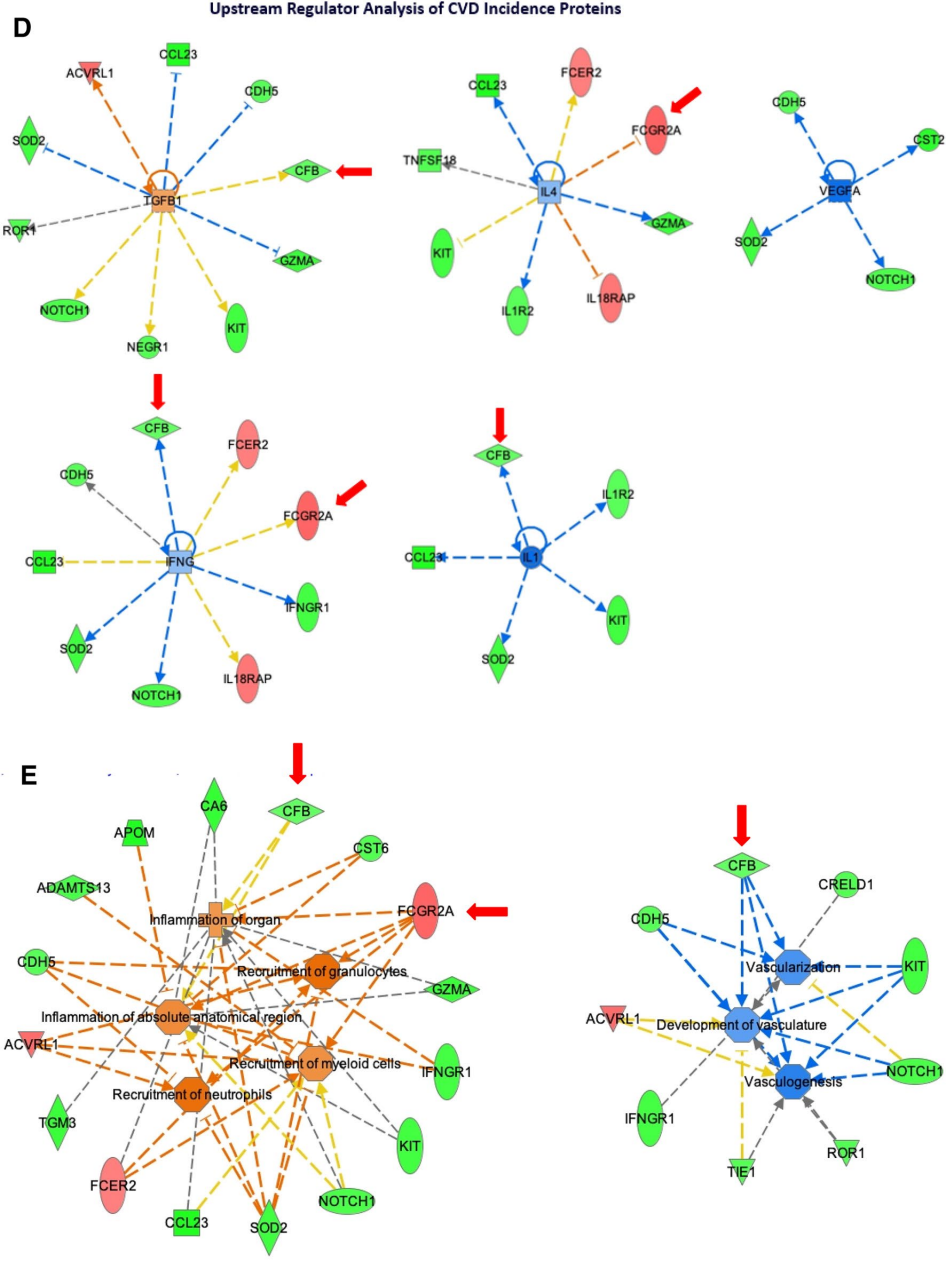
Systems Biology Analysis of the 36 CVD Incidence Proteins. (**A**) Interactive network analysis. Ingenuity Pathways Analysis was applied to generate the interactive networks from the 36 proteins associated with CVD events. The interactive network with the highest statistical significance is shown here. Red indicates protein up-regulation and green denotes protein down-regulation in individuals with CVD event. The intensity of the node color indicates the degree of up-regulation (red) and down-regulation (green) in individuals with a CVD event as compared with the matched controls. Empty shapes reflect proteins/genes not differentially expressed or absent from the SOMAscan platform that were brought in as interactors. Proteins are coded by shape. Square: cytokine; vertical rhombus: enzyme; horizontal rhombus: peptidase; trapezoid: transporter; ellipse: transmembrane receptor; circle: other. Links reflect various potential interactions such as protein expression regulation, protein activity, or modification of the other protein–protein interactions. Red arrows indicate the 3 proteins significant in final multivariate CVD-protein signature models. (**B**) Upstream Regulator Analysis of CVD Incidence Proteins. Upstream regulators (i.e., a protein/gene that can affect the expression of another protein/gene) with highest statistical significance that best explain the observed expression changes in the input 36 protein list as their targets. The x-axis indicates the -log p-values. (**C**) Diseases and Bio Functions Analysis of CVD Incidence Proteins. Biological functions that are significantly enriched (i.e., statistically relative high number of proteins associated with CVD by the 36-input protein list. The x-axis indicates the -log p-values. (**D**) Upstream Regulator Analysis of CVD Incidence Proteins. Downstream targets of upstream regulators (i.e., proteins whose expression is affected by TGFB1, IL4, VEGFA, IFNG, IL1) of the 36 CVD-associated proteins. Red indicates up-regulation and green denotes down-regulation in individuals with a CVD event. Proteins are coded by shape. Square: cytokine; vertical rhombus: enzyme; horizontal rhombus: peptidase; trapezoid: transporter; ellipse: transmembrane receptor; circle: other. Links are color-coded. Red: leads to activation; blue: leads to inhibition; yellow: findings inconsistent with state of downstream protein; black: effect not predicted. The red arrows indicate the 3 proteins significant in final multivariate CVD-protein signature models. (**E**) Diseases and Bio Functions Analysis of CVD Proteins. Proteins among the input list that are linked to immune functions (Left) and vascular functions (Right). The color, shape, and link coding are the same as part (**D**).

Ingenuity Pathway Analysis was performed using the 36 proteins significantly associated with CVD to obtain insights into enriched signaling pathways and biological mechanisms. Interactive network analysis generated a network of primarily inflammation and immune function associated proteins/genes that incorporated 24 of the 36 proteins into a single network, indicating a significant relationship among the majority of proteins associated with CVD incidence in our sample (Fig. 5A). Proteins up-regulated in CVD compared to controls are highlighted in red and down-regulated proteins in green.

We then modeled the links between differential expression of these 36 proteins based on their established associations with shared upstream regulatory proteins and biological effects. The upstream regulators and biological functions that were most significantly enriched by the 36 proteins are shown in Fig. 5B,C. Pro-inflammatory cytokines such as IL-1, IL-4, and IFNG are predicted upstream regulators of 5, 8, and 9 of the 36 CVD-associated proteins, respectively (Fig. 5D). Similarly, TGFB1 and VEGFA are predicted upstream growth factors regulating 10 and 4 of the 36 CVD-associated proteins, respectively (Fig. 5D). We also saw enrichment for enhanced recruitment of immune cells, such as granulocytes, neutrophils, and myeloid cells, as the most prominently enriched biological function, followed by reduced activity of vasculature functions (Fig. 5E). Figure 5E highlights in detail the proteins linked to immune cell recruitment and vascular functions among the 36 proteins. This figure demonstrates, for example, that recruitment of granulocytes or neutrophils is linked to a similar and overlapping set of proteins as can be seen by the connecting nodes leading from recruitment of granulocytes to mostly the same proteins as recruitment of neutrophils.

### Multivariable modeling of protein and clinical variables

From the 36 proteins that were statistically significant at the type-I error threshold of 0.05 in the univariable logistic regression, we performed stepwise model selection using Akaike Information Criterion (AIC) to arrive at a final multivariable model of CVD risk that included three proteins: Contactin-5 (CNTN5), Low affinity immunoglobulin gamma Fc region receptor II-a (FCGR2A), and Complement factor B (CFB). Figure 5 highlights these proteins (red arrows) in the context of the interaction and upstream regulator networks. Separately, we also modeled the associations between MASALA clinical variables and CVD. We considered the following variables for this model: age, sex, smoking, type 2 diabetes (T2D), hypertension, LDL, and HDL. We did not include CAC score because it was highly correlated with hypertension. In the multivariable logistic regression with these seven clinical variables, we obtained an AUC (area under the receiver-operating-characteristic curve) of 0.69, but only T2D showed a significant adjusted effect on CVD (OR 3.0; 95% CI 1.2–7.5). Thus in our final main effects model, we included both clinical information and proteomic data, using the following 4 predictors: CNTN5, FCGR2A, CFB, and T2D. Table 3 shows that this model had an AUC of 0.82. CNTN5 and CFB exhibited down-regulated effects, such that an increase of 1 SD in CNTN5 and CFB RFU levels would decrease the odds of CVD by 70% and 55%, respectively. FCGR2A exhibited an up-regulated effect, such that 1 SD of increase in this protein would increase the odds of CVD by more than threefold.

**Table 3:** Multivariable logistic regression modeling of effects of selected proteins on CVD incidence^*^.

Protein	Mean (SD) (RFU) Median (range) or prevalence	Adjusted OR (95% CI) by 1 SD increase in Protein	P-value
Contactin-5 (CNTN5)	331 (51) 321 (288–365)	0.30 (0.16–0.56)	0.0001
Low affinity immunoglobulin gamma Fc region receptor II-a (FCGR2A)	1272 (1322) 1152 (29–1771)	3.20 (1.63–6.19)	0.0007
Complement factor B (CFB)	22,613 (2604) 22,484 (21,272–24,258)	0.45 (0.26–0.77)	0.0036
Diabetes	37%	2.60 (0.97–6.98)	0.058
Area Under the ROC (AUC)	0.82		

*This multivariable model was selected from the 36 proteins that were statistically significant in the univariable model. The baseline clinical model of variables from Table 1 only included diabetes. The final main-effects model reported here includes these three proteins and diabetes.

### R/S associations with CVD

We assessed the influence of 10 different R/S measures or scales, as described in the Materials and Methods section, first assessing the direct association of these variables with CVD, and then assessing the modifying influence of these variables on the protein-CVD associations. With respect to associations between the R/S measures and CVD (Table 4), the largest absolute differences in CVD were seen according to positive religious coping (12%), religious struggles (12%), and Non-theistic Daily Spiritual Experiences (11%). There were moderate to high correlations among the R/S continuous variables. For example, positive religious coping and religious struggles were correlated with one another (Spearman correlation = 0.50), and both were correlated to Closeness to God at 0.80 and 0.35, respectively. Group prayer, individual prayer, and attendance at religious services or temple were correlated at above 0.40. Univariable logistic regression was used to assess the association between binary R/S variables (median- and conceptually-based) and CVD (the last 2 columns of Table 4). None showed statistically significant p-values; however, positive religious coping, religious struggles, and non-theistic daily spiritual experiences yielded the largest point estimates of odds ratios.

**Table 4:** Prevalence of R/S Variables and their Association with Incident CVD in the MASALA Study.

	Continuous* R/S Full sample^†^ (N = 100)	Median R/S^‡^ Full Sample (N = 100)	Conceptual^§^ R/S Full sample (N = 100)	Median-based R/S prevalence among cases (N = 50)	Median-based R/S Prevalence among controls (N = 50)	Median-based R/S univariable odds ratio (95% CI)^||^	Conceptual-based R/S univariable odds ratio (95% CI)^#^
Positive Religious Coping	2.78 (0.89)	42% (32)	51% (39)	48% (16)	36% (16)	1.65 (0.66–4.13)	1.31 (0.53–3.25)
Religious struggle	1.50 (0.71)	46% (36)	19% (15)	53% (18)	41% (18)	1.63 (0.66–4.01)	2.28 (0.72–7.20)
Closeness to God	4.21 (0.86)	50% (38)		52% (17)	49% (21)	1.11 (0.45–2.76)	
Group Prayer	2.49 (1.51)	37% (32)	31% (27)	35% (14)	38% (18)	0.87 (0.36–2.08)	0.91 (0.37–2.28)
Praying alone	5.11 (2.06)	20% (18)	72% (63)	20% (8)	21% (10)	0.95 (0.34–2.69)	0.87 (0.34–2.19)
Meditation	3.79 (2.09)	47% (41)		45% (18)	49% (23)	0.85 (0.37–1.99)	
Religious Service Attendance	3.77 (0.96)	20% (18)	63% (57)	18% (7)	22% (11)	0.75 (0.26–2.16)	0.64 (0.27–1.51)
Gratitude	4.63 (0.66)	65% (56)		67% (26)	64% (30)	1.13 (0.46–2.77)	
Non-theistic Daily Spiritual Experience	3.49 (0.71)	41% (35)	34% (29)	35% (13)	46% (22)	0.64 (0.27–1.55)	0.57 (0.22–1.43)
God Locus of Control	1.45 (0.59)	40% (31)	39% (29)	38% (13)	41% (18)	0.89 (0.36–2.24)	0.89 (0.36–2.34)

*Mean (Standard Deviation).

^†^Of the 100 subjects in the sample, depending on the R/S variable, 10 to 14 subjects do not have R/S data.

^‡^R/S variables dichotomized at the median of the distribution of the continuous R/S variables (yes: above median).

^§^R/S variables dichotomized into conceptually based R/S variables (yes: above threshold).

^||^Univariable logistic regression where Incident CVD is the dependent variable, and the Median-based R/S variable is the independent variable. This is the odds ratio of Incidence of CVD above versus below median of the R/S variable distribution.

^#^Univariable logistic regression where Incident CVD is the dependent variable, and the conceptually-based R/S variable is the independent variable. This is the odds ratio of Incidence CVD of above versus below conceptual threshold of the R/S variable distribution.

### Effect modification by measures of R/S

Because we hypothesize that R/S variables may be important resources for resiliency that modify the impact of stress on CVD, we then carried out effect modification analyses using the R/S variables. We hypothesized that R/S variables would decrease associations observed among participants between protein concentrations and increased risk of CVD, with the exception of religious struggles, which we anticipated would have the opposite effect (increasing the strength of protein-CVD risk associations). We used the above main effects model that included the three proteins and T2D for effect modification analyses. First, we added an interaction term between each protein and each median-based R/S variable (dichotomized above and below the median of the continuous R/S variable) to this model. Religious struggle had a significant interaction with CNTN5 (p-for-interaction: 0.009) and CFB (p-for-interaction: 0.004). We used linear contrasts to estimate the effect of protein levels (increase of 1 SD) on CVD for those above and below the median score for religious struggle (Table 5). The adjusted odds ratio for the effect of CNTN5 levels on CVD varied from 0.04 (religious struggle above median) to 0.24 (religious struggle below median). Contrary to our hypothesis, we found that while increasing protein levels of CNTN5 decreases the odds of CVD, this decrease in odds of CVD is even greater in those with high religious struggle (above median).

**Table 5:** Influence of Religious struggle on Associations between Proteins and Incident CVD Using Linear Contrasts^*^.

Protein	P-value of interaction term between protein and negative coping	Effect of 1 SD increase of protein for negative coping below median	Effect of 1 SD increase of protein for negative coping above median
Contactin-5 (CNTN5)	0.0085	0.24 (0.08–0.67)	0.04 (0.01–0.22)
Low affinity immunoglobulin gamma Fc region receptor II-a (FCGR2A)	0.5654	5.68 (1.76–18.37)	9.32 (1.88–46.26)
Complement factor B (CFB)	0.0044	0.18 (0.07–0.49)	0.86 (0.38–1.94)
Area Under the ROC (AUC)^†^	0.91		

*The linear contrasts came from the interaction terms of proteins and the median-based negative coping variable. Three interaction terms were included in the main effects model shown in Table 3. Shown are the odds ratios and 95% confidence interval for each stratum of negative coping.

^†^AUC from main effects model + interaction term of religious struggle x protein.

For CFB and CVD, however, the adjusted odds ratio varied in the opposite direction, in concert with our hypothesis, ranging from 0.86 (religious struggle above median) to 0.18 (religious struggle below median). This demonstrates that while increasing protein levels of CFB would decrease the odds of CVD, this decrease in odds of CVD is less in those reporting high levels of religious struggle (above median). For FCGR2A and CVD**,** where no significant interaction between the protein and reported levels of religious struggle was found, the adjusted odds ratio varied from 5.68 (below-median score for religious struggle) to 9.3 (above-median score for religious struggle).

It is significant to note that the AUC for the model with the interaction term for religious struggle (Table 5) is 0.91, a substantial improvement from the AUC of 0.82 (Table 3) in the model without the interaction term.

## Discussion

In this paper, we present a case–control proteomics analysis among 100 South Asian adults from the MASALA study who are also participating in the SSSH, in which we found significant associations of three proteins (CNTN5, CFB, and FCGR2A) with risk of a future CVD event. Specifically, an increase of 1 SD in RFU of CNTN5 and CFB would decrease odds of having a CVD event by 70% and 55%, respectively; an increase of 1 SD in RFU of FCGR2A would increase the odds of having a CVD event by 3.2-fold. We then tested our hypothesis that certain religious or spiritual (R/S) practices or beliefs would modify the effect of significant proteins on risk of having a future CVD event. Two of these protein-CVD associations (CNTN5 and CFB) were significantly modified by reported levels of religious struggle/spiritual struggles, a strategy for coping with stressful life situations in which an individual understands such events as proof that their God has abandoned them, that they are being punished for their transgressions, or some other negative religious belief.

Our finding that CFB protein is associated with risk of CVD echoes four previous studies, only one of which was conducted in a South Asian sample^33–36^. CFB has been shown to be elevated in adipose tissue and serum from South Asians with type 2 diabetes and CVD^37^, to correlate with fasting glucose and circulating lipids^38^, and to increase the risk of heart disease^39^, but the relationship of CFB to disease is still not fully understood^36^. It is unclear why our analysis showed an effect in the opposite direction, where increased CFB protein was associated with lower risk of CVD.

There is less existing research assessing the associations of CNTN5 and FCGR2A proteins with risk of CVD, although there is some evidence suggesting that polymorphisms in their corresponding genes may play a role in CVD. In particular, a polymorphism in the *CNTN5* gene has been associated with atrial fibrillation and heart failure in a genome-wide analysis of the (mostly white) Framingham Heart Study participants^33^. Knockout of the *CNTN5* gene in mice has also been shown to elevate several cardiovascular parameters, including heart rate, blood pressure, and blood flow speed^40^. A polymorphism in the *FCGR2A* gene has also been shown to predict coronary artery disease, and was also associated with altered levels of C-reactive protein^41^. While decreased FCGR2A expression has been reported to play a role in the pathogenesis of atherosclerosis, increased FCGR2A expression in platelets may also play a role in atherothrombosis^41,42^.

Based on public databases such as Human Protein Atlas (https://www.proteinatlas.org) and GeneCards (https://www.genecards.org), all 3 of our significant proteins (CNTN5, CFB, FCGR2A) are expressed under various conditions in multiple bodily fluids, organs, and cell types. This is not unexpected, as this is case for the majority of human proteins. Furthermore, several previous SOMAscan studies have identified these 3 proteins as differentially expressed in various non-CVD disease phenotypes. This includes a published study on ovarian cancer, in which SOMAscan data were compared to antibody-based Olink, demonstrating that CNTN5 is significantly differentially expressed and highly correlated between the 2 platforms^43^. A second Alzheimer’s disease exosome study incorporated CNTN5 into a panel that predicts Alzheimer’s, with a high AUC^44^. And lastly, another Alzheimer’s disease study has also demonstrated differential expression of CFB in relation to Alzheimer’s^45^.

Systems biology analysis of the 36 CVD-associated proteins, including the 3 proteins selected for our multivariable analysis, suggested that the majority of these proteins are interconnected in an interactive network downstream of several cytokines and growth factors and relate to immune cell recruitment and vascularization. This list of 36 proteins includes several proteins previously linked to CVD, such as ADAMTS13 and LRIG3. Lower levels of plasma ADAMTS13 have been demonstrated to be associated with CVD in young patients^46^ and to predict cardiovascular events in type 2 diabetics^47^. LRIG3 has been shown to increase blood pressure and induce cardiac hypertrophy^48^. There are a number of published studies that link circulating blood or urine proteins to CVD, including several studies utilizing the SOMAscan platform^49–52^. Most notably, a recent, large-scale study using SOMAscan among participants with stable coronary heart disease (CHD) in the Heart and Soul Study (N = 938), and a validation analysis in the HUNT3 study (N = 971), identified a 9-protein risk score that was derived and validated for 4-year probability of myocardial infarction, stroke, heart failure, and all-cause mortality^27^. These nine proteins included ANGPT2, MMP12, CCL18, C7, SERPINA3, ANGPTL4, TNNI3, GDF8/11, SERPINF2. This 9-protein risk score did not include any of the 3 proteins discovered in our study. However, these previous studies included, for the most part, white populations of relatively high-risk individuals with established CVD. Our analysis, on the other hand, was conducted in persons with and without a CVD event among U.S South Asians, who have not been included in extant studies. It is essential that biomarker research aimed at improving CVD prevention includes participants from minority racial and ethnic communities that bear a disproportionate burden of CVD. Although U.S. South Asians bear a higher burden of CVD compared with other U.S. populations^1^, no proteomics research has been conducted in this sub-population prior to our study. If proteomics research is to inform efforts to reduce racial/ethnic disparities in the burden of CVD, research to identify protein biomarkers must include adequate numbers of individuals from communities that bear the highest rates of CVD-related morbidity and mortality.

Our analysis is also the first exploration of whether R/S influence proteomic profiles associated with risk of CVD. Although religiosity and religious service attendance have been associated with incident CVD in a large prospective cohort study and several meta-analyses^8,19,20^, to date no R/S measures have ever been investigated in a proteomic analysis. Our results are the first to demonstrate that religious struggles significantly influence the impact of protein concentrations on risk of CVD. In our sample of U.S. South Asians, among those who reported religious struggles (e.g., feeling they were being punished by God, abandoned by God, doubting their faith) in response to stressful life events, 1 SD increase in CFB concentration was associated with a smaller decrease in CVD risk (14%) relative to those that did not employ religious struggle (82%). This may be in part because religious struggles may be stressful events in and of themselves. In this case, it is plausible that stress from religious struggles has a stronger effect on risk of CVD than CFB concentrations, thereby cancelling out the positive impact of greater concentrations of CFB. Religious struggles have not been investigated in relationship to CVD risk, but have been shown in previous studies to associate with stress, inflammation, and immune markers such as cortisol^53^, CD4 levels^54^, and interleukin-6^55^.

It is curious, however, that our results show religious struggles having the opposite effect on the association between CNTN5 and CVD risk, even though both CFB and CNTN5 were both shown to decrease risk of CVD in our analysis. Given that the biological pathways through which religious struggles work to affect health are still largely unknown, and that CNTN5 is also a protein about which we have relatively little mechanistic knowledge in the context of CVD, it is difficult to disentangle these relationships. Notably, the researcher who developed our religious coping measure has stated that religious struggles should not be considered universally maladaptive, and that “the efficacy of particular coping methods is determined by the interplay between personal, situational, and social-cultural factors, as well as by the way in which health and well-being are conceptualized and measured”^16^. The self-reported and somewhat subjective nature of these questionnaire items makes interpretation of such factors less straightforward than for biological variables. These pilot study results should be taken as preliminary associations that warrant further investigation in larger samples that can support more robust modeling in order to confirm the magnitude and the direction of the impact of religious struggle and other R/S measures on these significant protein-CVD associations across South Asian and other understudied racial/ethnic communities. These future studies will need to pay careful attention to modeling the interplay of R/S with the personal, situational, and socio-cultural factors mentioned above.

Notably, our analyses only include one protein measurement from a single time point for each subject; therefore, there is clearly a concern regarding the reproducibility and stability of these protein measurements. We were constrained by limited resources to run the SomaScan assay more than once for each MASALA participant, however we attempted to evaluate this issue by checking the stability of protein levels in seven participants that we are studying in another cohort, the Nurses Health Study II (NHSII), who have SomaScan data at two time points (baseline and 1-year). We looked at the same three proteins (CNTN5, FCGR2A, CFB) and assessed the stability of these proteins across the two time points in our NHSII participants, one year apart. Our results indicate that the levels of these three proteins remain quite similar between the two time points (Supplemental Table 1). Compared to these three proteins in our case–control study, the range of CFB is of similar magnitude between the NHSII data and our data. FCGR2A in these NHSII patients falls in the range of FCGR2A for our data. CNTN5 in the NHSII study for these patients is similar to CNTN5 distribution in our data. Overall, it does appear that these proteins are stable over time, at least in the observed 1-year span seen here in the NHS data.

Although our study data show robust discovery of three candidate biomarkers associated with CVD and religious struggle, discovery studies need to be paired with robust validation in order to establish quantitative accuracy for deleterious levels of protein concentrations affecting risk of CVD^23^. We plan to follow up this pilot study with a larger validation study in the context of the diverse participants from the SSSH, which includes approximately 1000 Black, Hispanic/Latino, American Indian, South Asian, and white participants, for a total of ~ 5000 participants. Our pilot study also comprised a relatively small sample size, although we demonstrate that we had adequate statistical power for all analyses (see “Methods”). Despite these limitations, this pilot study appears to be the first untargeted proteomics investigation of proteins that predict incident CVD in a U.S. South Asian population, and the first to investigate the role of R/S in modifying these relationships in any population. Our results provide a strong rationale for further investigation of these proteins as predictive protein markers of CVD in this high-risk ethnic population, and potentially other minority communities. Our results also provide a strong rationale for further investigating the biological impact R/S beliefs and practices may have on protein expression and risk of CVD, whether exerting a protective or deleterious effect, in order to creatively explore novel avenues for reducing the burden of CVD and disparities in the burden of illness.

## Materials and methods

### Study population

This analysis used data from a subsample of participants from the Mediators of Atherosclerosis in South Asians Living in America (MASALA) Study who are also participating in the Study on Stress, Spirituality, and Health (SSSH). The SSSH is a national multi-cohort study that brings together data from racially and ethnically-diverse cohorts and collects new data on all SSSH participants through periodic surveys to understand the relationships between psychosocial stress, religious and spiritual (R/S) practices and beliefs, and health. MASALA is an ongoing study designed to investigate causes of cardiovascular disease among South Asians not explained by traditional risk factors. In addition to CVD, MASALA measured a number of other facets of psychosocial and behavioral health. Participants were initially recruited from 2010 to 2013^56^. To be eligible, participants must have been between 40 and 84 years of age, of South Asian descent, free of cardiovascular disease, and able to speak, read, and write in English, Hindi, or Urdu. Recruitment took place in the San Francisco Bay and greater Chicago areas using telephone-based recruitment methods in areas where census data revealed high proportions of South Asians; 906 original cohort members (Exam 1) underwent a 6-h baseline examination.

During a follow-up visit of the MASALA study between 2016 and 2018 (Exam 2), 733 returning cohort members completed the baseline Spirituality Survey (SS-1) of the SSSH. A new recruitment wave from 2017–2018 (Exam 1A) resulted in an additional 257 participants who completed both a MASALA baseline examination and the SSSH SS-1. In all, 990 MASALA participants completed the SS-1 at baseline and follow-up visit.

We used a nested case–control design with a total of 100 MASALA participants who completed the SS-1. All subjects ranged in age from 40–84 at the time of blood collection (either at Exam 1, 2010–2013 or Exam 1A, 2017–2018). Fifty cases were selected who had reported a CVD event by June 2018 and were matched by age and sex to a random sample of 50 participants who did not report any CVD events by the censoring time. CVD events were confirmed by medical records review (adjudicated by 2 physicians) and completed all R/S measures to be used in this analysis.

### Specimen collection

Fasting blood samples were obtained during the baseline MASALA clinical visit (2010–2013 or 2017–2018) by a trained phlebotomist. EDTA plasma was isolated and stored at − 80 °C in the MASALA core research lab. Samples were pulled and shipped to the Genomics, Proteomics, Bioinformatics and Systems Biology Center at Beth Israel Deaconess Medical Center in Boston, Massachusetts for analysis.

### CVD cases

Cases were defined as having a myocardial infarction, stroke or transient ischemic attack, and/or revascularization (coronary stent, coronary artery bypass graft surgery, or carotid endarterectomy).

### Clinical covariates

CVD variables and clinical covariates were assessed at the follow-up MASALA clinical visit (2015–2018) using clinical measurement. Clinical covariates used were age, sex, body mass index (BMI), diagnosis of type 2 diabetes (T2D), diagnosis of hypertension, LDL-cholesterol, HDL-cholesterol, coronary artery calcium (CAC) score, and smoking.

### R/S variables

We included 10 R/S measures in our analyses, including 5 R/S scales and 5 individual R/S items. All items were taken from the SS-1 and prefaced with the following statement: “These questions are being asked of people from different religious backgrounds, and although we use the term ‘God’ in some of the questions below, please substitute your own word for ‘God’ (for example, Bhagwan, Allah, The Divine, etc.)”.

#### Positive coping and religious struggles

Two subscales included eight positive religious coping items (α = 0.93) and six religious struggles items (α = 0.77) from the RCOPE^57^, with input from the scale creators in reducing the number of sub-items assessed. All religious coping items were completed in response to the prompt, “In facing recent stressful life events…” Positive religious coping items included: “I saw my situation as part of God’s plan,” “I tried to see how God might be trying to strengthen me in these situations,” “I tried to make sense of the situation with God,” “I worked together with God to relieve my worries,” “I did what I could and put the rest in God’s hands,” “I took control over what I could, and gave the rest up to God,” “I sought God’s love and care,” and “I trusted that God would be by my side.” Religious struggle items included: “I wondered what I did for God to punish me.” “I wondered if God allowed this event to happen to me because of my sins,” “I believed the devil or evil spirits were responsible for my situation,” “I felt as though the devil or an evil spirit was trying to turn me away from God,” “I wondered whether God had abandoned me,” “I questioned God’s love for me.” Response categories include 1: not at all, 2: somewhat, 3: quite a bit, 4: a great deal. Items were averaged to create measures of positive and religious struggle.

#### Closeness to god (4 items)

Among those who indicated belief in God, four items assessed participants’ perception of their relationship with their God: “God gives me the strength to do things I otherwise could not do myself,” “God loves or cares for me unconditionally, in a way I could never earn,” “Throughout my life, God has come through for me,” “God is the center of my life,” and “When I pray, I feel a deep sense of closeness with God” (all de novo). Each ranged from 1 to 5 (1: definitely not true of me, 2: tends not to be true of me, 3: unsure, 4: tends to be true of me, 5: definitely true of me). Responses were averaged to create a Closeness to God scale.

#### Religious and spiritual practices (4 items)

Four separate items assessed frequency of: religious service attendance, group prayer outside of service attendance, praying alone, and meditation, with responses ranging from 1 (never) to 7 (several times a day). Each of these measures was evaluated individually.

#### Gratitude (2 items)

Two questions assessed feelings of gratitude (α = 0.80), drawn from the Gratitude Questionnaire-6^58^: Responses to these two items were averaged to create a measure of gratitude.

#### Non-theistic daily spiritual experiences (4 items)

Non-theistic subscales of the Daily Spiritual Experiences Scale (DSES)^59^ were used to assess spiritual experiences that do not depend on belief in God. Four items formed the DSES non-theistic subscale: “I experience a connection to all of life,” “I feel deep inner peace or harmony,” “I am touched by the beauty of nature,” and “I feel a selfless caring for others.” Responses ranged from “never” to “many times a day.” Responses were averaged to create a non-theistic DSES scale.

#### God locus of control (1 item)

A single item was used to assess participants’ beliefs regarding the extent to which their health is determined by their own actions or determined by their God: “When you think about God in relationship to your health, which of the following is closest to your own view?” Responses categories were: 1: my health is determined by my own actions and behaviors, 2: when it comes to my health, God and I both have a role to play, 3: [God] determines my health, regardless of my own actions and behaviors.

All R/S scales and measures were converted to binary variables for analysis. For all variables except for closeness to God, gratitude, and meditation, binary variables were created in two separate ways: each R/S continuous (or ordinal) variable was dichotomized as above or below a conceptually-based threshold (“conceptually-based”), based on previous research, and then a separate set of binary variables were created by dichotomizing according to the median of the variable (“median-based”).The conceptual binary variables were created as follows: respondents were coded as having positive religious coping if they scored ≥ 3 on the scale (i.e., “quite a bit” or more). Religious struggle was less common, so respondents were coded as having religious struggle if the scale score was ≥ 2 (i.e., “somewhat” or more). Group prayer was relatively uncommon, so if respondents reported this activity once a week or more they were coded as someone who participates in group prayer. Individual prayer was more common, so participants were coded as those who regularly pray if they prayed privately once per day or more. Participants were considered to attend religious services/temple if they attended 2–3 times a month or more. Participants were considered to have non-theistic daily spiritual experiences if they scored ≥ 4 (“every day or more”) on the scale. Participants were considered to have God involved with their health locus of control if they answered either “my health is determined by my own actions and behaviors,” or “when it comes to my health, God and I both have a role to play.” Otherwise, they were coded as God not being involved in health locus of control.

Closeness to God, gratitude, and meditation variables were only dichotomized according to the median response value due to a lack of theoretical or empirical information to guide a conceptual threshold for dichotomization.

### SOMAscan assay

EDTA plasma samples (50 μl) from 100 individuals matched on age and gender were analyzed using the highly multiplexed SOMAscan Assay Kit for human plasma 1.3 k, which measures expression of 1,305 human proteins using highly selective single-stranded modified SOMAmers according to the manufacturer’s standard protocol (SomaLogic; Boulder, CO). The majority of the proteins measured by SOMAscan are known to be secreted, leaked, or shed from cells into the circulation^60^, making this proteomics platform an ideal technology for biomarker discovery in plasma. Five pooled plasma controls and one no-protein buffer control were run in parallel with the plasma test samples for each run. Sample to sample variability is further controlled by several hybridization controls. Each individual protein concentration was transformed into a corresponding SOMAmer concentration, then quantified using a custom DNA microarray (Agilent) read-out, which reports the data as relative fluorescence units (RFU)^29,61,62^. Data quality control, calibration, and normalization was done according to the manufacturer’s protocol as previously described^30^.

### Statistical analysis

#### Power analysis

As this is a pilot study, our power analysis was based on being able to identify several proteins that exhibit a significant signal in their association with CVD, and to assess effect modification from R/S variables of interest. We assumed that two models will be compared: the basic baseline clinical variables without a proteomic signature (MODEL 1), and one with protein predictors (MODEL 2). We examined the AUC for the baseline model (MODEL 1) of incident CVD that adjusts for age, sex, smoking, diabetes, hypertension, LDL, and HDL. We hypothesized that adding in the proteomic data would increase the predictive power of the model. Thus, “MODEL2 = MODEL1 + Proteins” is expected to have improved the aROC to a level of 0.80. Given a type-I error of 0.05, we would have power of 0.88 to detect the improvement of aROC from 0.70 (MODEL 1) TO 0.80 (MODEL 2) using a sample size of 100 participants^63,64^. The estimate aROC of 0.70 of the baseline model was based on the reported range from Alaa et al.^65^.

#### Statistical methods

For bivariate analyses, we used chi-square or Fisher’s exact test, and t-test or Wilcoxon Rank Sum test. We used nonparametric Spearman correlations to obtain pairwise correlations for all 1305 SOMAscan proteins, and between outcome and baseline clinical variables.

We used univariable logistic regression models to screen for the selection of the individual proteins. We used multivariable logistic regression for model selection using Akaike Information Criterion (AIC), to select the optimal model with both clinical information and selected proteins.

#### Effect modification

In the final main effect multivariable model (that included both clinical information and select proteins), we evaluated each interaction term between the selected protein and the selected R/S variable (each model was run separately for each R/S variable and its interaction with the selected proteins). Both the median-based and conceptually-based R/S variables were tested. We then used linear contrasts from the main effect (proteins) and the interaction term (protein*R/S variable) to obtain the estimated effect of an increase of 1 standard deviation of protein on incident CVD for each level (above or below median) of the R/S variable. We report the c-statistic or AUC of the final main effect model, as well as the model with effect modification. We conducted all statistical analyses using SAS/STAT software version 9.

### Systems biology analysis

Systems biology analyses of CVD-associated proteins were performed using the Ingenuity Pathways Knowledge Base (Qiagen, Redwood City, CA), a repository of biological interactions and functions created from millions of individually modeled relationships ranging from the molecular (proteins, genes) to organism (diseases) level. Ingenuity Pathway Analysis (IPA) uses enrichment analysis-based approaches^66,67^ to calculate the significance of observing a candidate protein set within the context of biological systems.

Ingenuity Pathways Analysis (IPA) has generated a knowledge database that defines and incorporates many different categories and subcategories of biological processes and disease functions based on gene/protein expression or interactions. Each function defined by IPA includes a specific set of genes/proteins that have been linked to the particular functional category based on published data. IPA then calculates the enrichment of genes/proteins from the list of genes/proteins in the particular test set to the genes/proteins included in each functional category and calculates the p-value for enrichment or overlap between the test set and the IPA knowledge base using Fisher’s Exact test.

The different functional categories are defined by IPA based on genes/proteins associated with such functions and can be displayed in a tree map which clusters related functions together, thus providing a high-level view of the function families. Consequently, there may be various related functions such as “recruitment of neutrophils” and “recruitment of granulocytes” that have many overlapping genes/proteins, but have also some that are unique to one but not the other. Since granulocytes includes basophiles, eosinophils, and neutrophils, granulocytes would be a higher-level hierarchical category than neutrophils and neutrophils would be a subcategory of granulocytes.

### Ethics statement

Institutional Review Board (IRB) approval for this study was obtained from the Partners Human Research Committee (PHRC) and the University of California, San Francisco Review Board.

## Supplementary Information

Supplementary Information

## Data Availability

MASALA study datasets may be obtained from the cohort’s Coordinating Center upon approval of an ancillary study proposal, signing a data use agreement, and obtaining IRB or other relevant ethics approval. The SS-1 data may be obtained from the SSSH Steering Committee after approval of an ancillary study proposal, signing of a data use agreement, and obtaining IRB or other ethics approval.
