# Supplementary material for: Plasma protein expression profiles, cardiovascular disease, and religious struggles among South Asians in the MASALA study
Source: Sci Rep. 2021 Jan 13;11:961. doi: 10.1038/s41598-020-79429-1 (PMC7806901; doi:10.1038/s41598-020-79429-1)
Supplement: Supplementary file 1 — Supplementary Information [file 41598_2020_79429_MOESM1_ESM.docx]

**Supplementary Materials**

**Plasma protein expression profiles, cardiovascular disease, and religious struggles among South Asians in the MASALA study**

Long H. Ngo^1,2,3*^, M. Austin Argentieri^4,5^, Simon T. Dillon^1,2^, Blake Victor Kent^4,6^, Alka M. Kanaya^7^^, Alexandra E. Shields^1,4^^, Towia A. Libermann^1,2^^

^1^ Harvard Medical School, Harvard University

^2^ BIDMC Genomics, Proteomics, Bioinformatics, and Systems Biology Center, Department of Medicine, Beth Israel Deaconess Medical Center

^3^ Department of Biostatistics, Harvard T.H. Chan School of Public Health

^4^ Harvard/MGH Center on Genomics, Vulnerable Populations, and Health Disparities, Mongan Institute, Department of Medicine, Massachusetts General Hospital

^5^ School of Anthropology and Museum Ethnography, University of Oxford

^6^ Department of Sociology, Westmont College

^7^ Department of Medicine, University of California, San Francisco (UCSF)

^ Co-senior authors

* Corresponding author:

Long H. Ngo

1309 Beacon Street, Brookline MA 02447

lngo@bidmc.harvard.edu

(617) 754-1415

**Supplemental Table 1**: Stability of protein concentrations (CNTN5, FCGR2A, CFB) among seven NHSII study participants at baseline and 1-year.


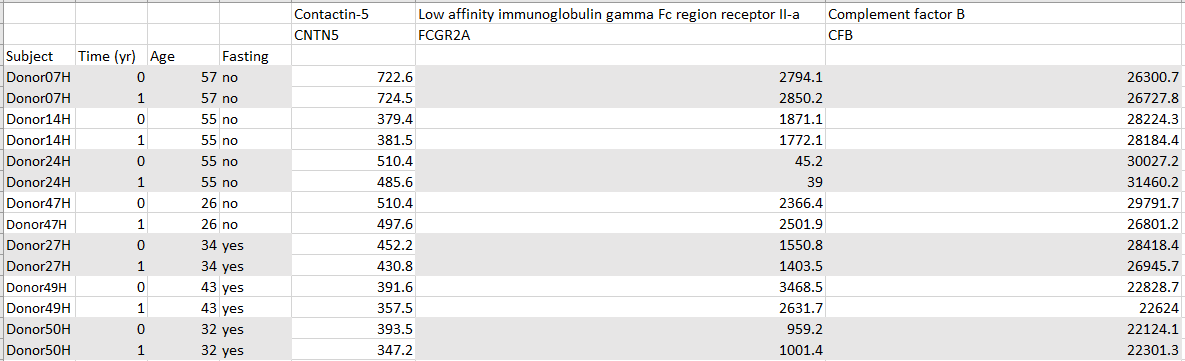


*Protein level measurements at two timepoints for a small subset of participants (n=7) from the Nurses Health Study’s II (NHSII) at baseline and one year later. We looked at the same three proteins (CNTN5, FCGR2A, CFB) from our analysis, and assessed the stability of the proteins across the two timepoints, one year apart. The table shows these data. Notice that the protein levels stay quite similar between the two timepoints. Compared to these three proteins in our case-control study, the range of CFB is of similar magnitude between the NHSII data and our data. FCGR2A in these NHSII patients falls in the range of FCGR2A for our data. CNTN5 in the NHSII study for these patients is similar to CNTN5 distribution in our data. Overall, it does appear that these proteins are stable over time, at least in the observed 1-year span seen here in the NHSII data.*
